# Preclinical characterization of tunlametinib, a novel, potent, and selective MEK inhibitor

**DOI:** 10.3389/fphar.2023.1271268

**Published:** 2023-09-21

**Authors:** Yahong Liu, Ying Cheng, Gongchao Huang, Xiangying Xia, Xingkai Wang, Hongqi Tian

**Affiliations:** Shanghai Kechow Pharma, Inc., Shanghai, China

**Keywords:** mek inhibitor, RAF/RAS mutant cancer, tunlametinib, drug combination, high potency

## Abstract

**Background:** Aberrant activation of RAS-RAF-MEK-ERK signaling pathway has been implicated in more than one-third of all malignancies. MEK inhibitors are promising therapeutic approaches to target this signaling pathway. Though four MEK inhibitors have been approved by FDA, these compounds possess either limited efficacy or unfavorable PK profiles with toxicity issues, hindering their broadly application in clinic. Our efforts were focused on the design and development of a novel MEK inhibitor, which subsequently led to the discovery of tunlametinib.

**Methods:** This study verified the superiority of tunlametinib over the current MEK inhibitors in preclinical studies. The protein kinase selectivity activity of tunlametinib was evaluated against 77 kinases. Anti-proliferation activity was analyzed using the 3-(4,5-dimethylthiazole-2-yl)-2,5-diphenyl-2H-tetrazolium bromide (MTT) or (3-(4,5-dimethylthiazol-2-yl)-5-(3-carboxymethoxyphenyl)-2-(4-sulfophenyl)-2H-tetrazolium) (MTS) assay. ERK and phospho-ERK levels were evaluated by Western blot analysis. Flow cytometry analysis was employed to investigate cell cycle and arrest. Cell-derived xenograft (CDX) and Patient-derived xenograft (PDX) models were used to evaluate the tumor growth inhibition. The efficacy of tunlametinib as monotherapy treatment was evaluated in *KRAS/BRAF* mutant or wild type xenograft model. Furthermore, the combination studies of tunlametinib with BRAF/KRAS^G12C^/SHP2 inhibitors or chemotherapeutic agent were conducted by using the cell proliferation assay *in vitro* and xenograft models *in vivo*.

**Results:** *In vitro*, tunlametinib demonstrated high selectivity with approximately 19-fold greater potency against MEK kinase than MEK162, and nearly 10–100-fold greater potency against *RAS/RAF* mutant cell lines than AZD6244. *In vivo,* tunlametinib resulted in dramatic tumor suppression and profound inhibition of ERK phosphorylation in tumor tissue. Mechanistic study revealed that tunlametinib induced cell cycle arrest at G0/G1 phase and apoptosis of cells in a dose-proportional manner. In addition, tunlametinib demonstrated a favorable pharmacokinetic profile with dose-proportionality and good oral bioavailability, with minimal drug exposure accumulation. Furthermore, tunlametinib combined with BRAF/KRAS^G12C^/SHP2 inhibitors or docetaxel showed synergistically enhanced response and marked tumor inhibition.

**Conclusion:** Tunlametinib exhibited a promising approach for treating *RAS/RAF* mutant cancers alone or as combination therapies, supporting the evaluation in clinical trials. Currently, the first-in-human phase 1 study and pivotal clinical trial of tunlametinib as monotherapy have been completed and pivotal trials as combination therapy are ongoing.

## 1 Introduction

Cancer incidence and mortality are rapidly increasing worldwide, and cancer is expected as the sole most important barrier to increasing life expectancy globally. Estimates of 19.3 million new cases and 10.0 million cancer deaths worldwide in 2020 were reported by World Health Organization (Sung et al., 2021; World Health Organization, 2022). Mitogen-activated protein kinase (MAPK)/extracellular regulated protein kinases (ERK) signaling pathway (also known as RAS/RAF/MEK/ERK signaling pathway) plays a critical role in cancer cell proliferation and apoptosis. Aberrant activation of this signaling pathway has been implicated in more than one-third of all malignancies. In this pathway, activated RAF phosphorylates and activates MEK1 (mitogen-activated protein kinase kinase) and MEK2 kinases, leading to downstream phosphorylation and activation of extracellular signal-regulated kinases, ERK1 and ERK2, which in turn triggers downstream activation of nuclear and cytoplasmic targets associated with transcription, cell proliferation, differentiation and metabolism (Pylayeva-Gupta et al., 2011). Approximately 20% of all human cancers have *RAS* gene activating mutations (Prior et al., 2020), including carcinomas of the lung (30%), colon (50%) and pancreas (90%), thyroid (50%), and melanoma (25%) (Diaz-Flores and Shannon, 2007; Roberts and Der, 2007). Mutation of *BRAF* is present in 7%–10% of all human cancers, while mutated forms of *ARAF* and *CRAF* are extremely rare (Yaeger and Corcoran, 2019).

MEK inhibitors target RAS/RAF/MEK/ERK signaling pathway in both *RAS* and *RAF* mutant genotypes (Solit et al., 2006). To date, four MEK inhibitors, including trametinib (also known as GSK212), cobimetinib, binimetinib (also known as MEK162), and selumetinib (also known as AZD6244), have been approved by U.S. Food and Drug Administration (FDA). Despite progress has been achieved in targeting this signaling pathway, current therapeutic approaches are not efficacious enough in a broad range of cancers. In addition, acquired drug resistance in clinic application is common during targeted therapy. Remarkable progress has been achieved over decades in understanding the profile of MEK inhibitors (Cheng and Tian, 2017). Current MEK inhibitors show either unsatisfactory potency or unfavorable pharmacokinetics (PK) profile with toxicity issue. Of which, selumetinib possesses an unsatisfactory potency *in vitro* and *in vivo*. This probably caused the failure of Randomized Clinical Trial for *KRAS*-mutant non-small cell lung cancer (NSCLC) without significant improvement in progression-free survival (Jänne et al., 2017). Likewise, binimetinib and REDA119 possess moderate potency (Cheng et al., 2019), with IC_50_ range of 30–250 nM (Tran and Cohen, 2020) for *BRAF-* and *NRA*S-mutant cell lines, and 19 nM/47 nM for MEK1/2 kinase (Iverson et al., 2009), respectively. Cobimetinib inhibited hERG channel with IC_50_ of 0.5 μM, suggesting some potential for causing corrected QT (QTc) prolongation *in vivo* (Food and Drug Administration, 2015). Trametinib has a long circulating half-life, resulting in nearly 6-fold drug accumulation (Infante et al., 2012). Hence, novel compounds targeting this pathway without causing unacceptable levels of toxicity are required. To our knowledge, the unfavorable profiles of current MEK inhibitors are closely correlated with their molecular structures. Of these, the interaction force of F- located in benzene ring of cobimetinib as H-acceptor is too weak to closely bind with MEK kinase, resulting in unsatisfactory potency. Trametinib shows poor solubility property and requires DMSO as co-solvate, resulting in drug accumulation in body and toxicity issue (Infante et al., 2012). Selumetinib and binimetinib share a similar structure, possess satisfactory PK profile, but need enhance efficacy (Cheng et al., 2019). Design and development of new MEK inhibitors with improved response and reduced toxicity represents new opportunities to confer effective therapy benefits for *RAS/RAF* mutant cancers.

**FIGURE 1:**
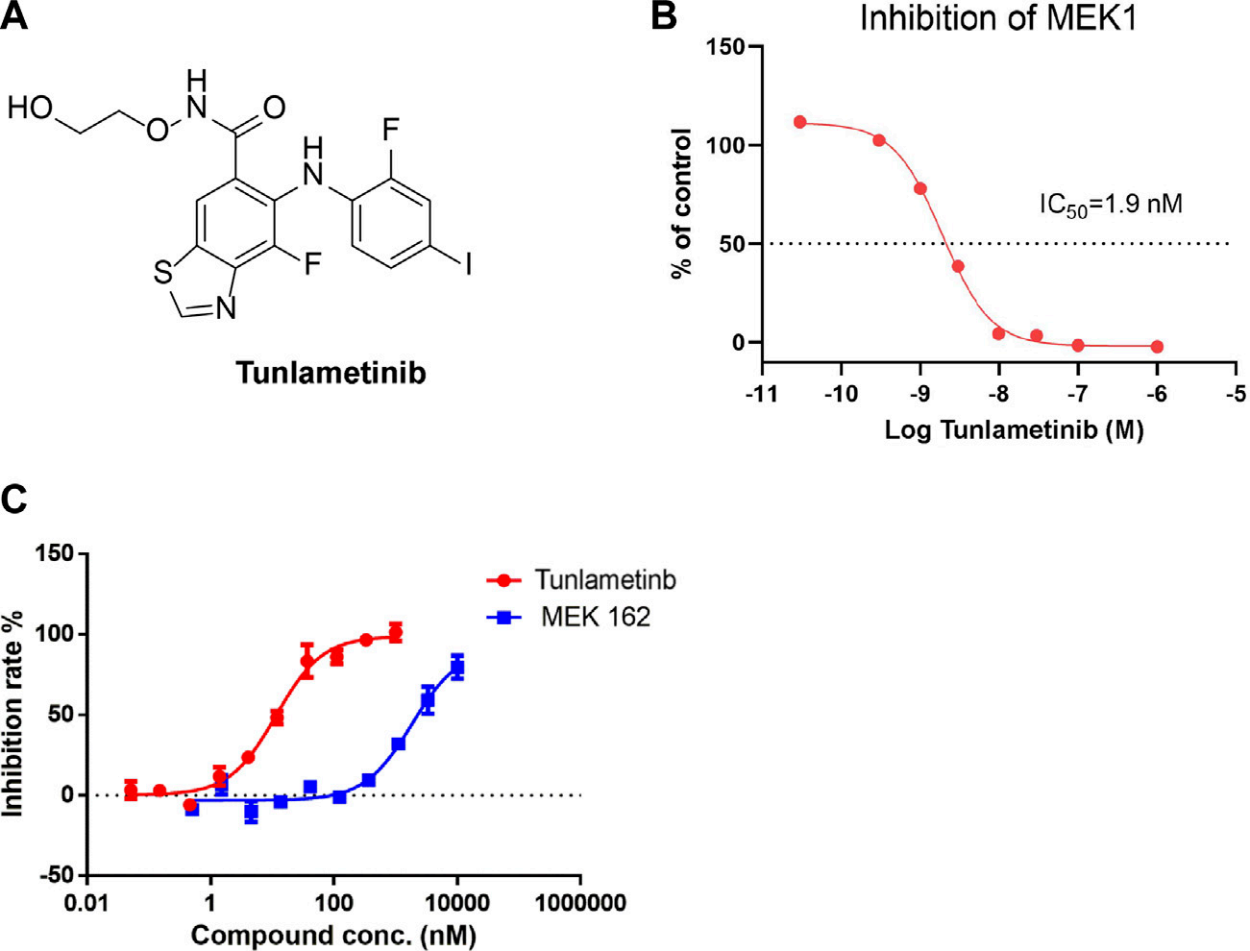
Inhibition curve of tunlametinib on MEK1/MAP2K1 (H) kinase. **(A)** Chemical structure of tunlametinib. **(B)** Tunlametinib on MEK1/MAP2K1 (H). Tunlametinib demonstrated a strong inhibition against MEK1/MAP2K (H) kinase with IC_50_ value of 1.9 nM. **(C)** *In vitro* kinase inhibition curves of tunlametinib and MEK162. Compared to MEK 162, tunlametinib showed stronger inhibitory activity, with IC_50_ value of 12.1 ± 1.5 nM versus 223.7 ± 16.9 nM.

Driven by the clinical need, our efforts were focused on developing a new MEK inhibitor with enhanced efficacy and favorable PK profile. Retrospective analysis showed that a strong hydrogen-bond interaction between MEK inhibitors and S212 in MEK allosteric pocket results in a superior antitumor efficacy in KRAS-driven tumors as it is critical for blocking MEK feedback phosphorylation by wild-type RAF (Hatzivassiliou et al., 2013). The aromatic nitrogen (N) of binimetinib forms a stronger hydrogen bond interaction with S212 binding site than aromatic fluorine (F) of cobimetinib, which means binimetinib has a higher potency in KRAS-driven cancers than cobimetinib. In addition, unlike trametinib, binimetinib has an exclusive binding mode that does not block binding and phosphorylation by Raf, thereby permitting incredible selectivity of MEK1/2. Hence, the well-known MEK inhibitors binimetinib was chosen as a lead compound under comprehensive analysis. As analyzing the key interactions between MEK inhibitors and the MEK allosteric pocket, a structurally new MEK inhibitor, designated as tunlametinib, was discovered (Figure 1A). Tunlametinib exhibited both enhanced efficacy and favorable PK profile in preclinical study, thereby overcoming the shortcomings of the current MEK inhibitors. In the phase 1 study of tunlametinib monotherapy for NRAS-mutant melanoma, tunlametinib demonstrated acceptable tolerability and substantial clinical activity as well as favorable PK profiles (Zhao et al., 2022; Wang et al., 2023). The encouraging efficacy and well tolerability were further verified in a phase 2 trial (Si et al., 2023). Herein, we describe the preclinical characterization of tunlametinib.

Combination therapies attempt to improve therapeutic responses and reduce the likelihood of acquired resistance in cancer patients (Palmer and Sorger, 2017). Combination therapy with MEK plus BRAF inhibitors could improve therapeutic outcomes in *BRAF*-mutated cancers and delay or prevent drug resistance, also be superior to monotherapy in terms of efficacy without significant increase in toxicity (Richman et al., 2015; Subbiah et al., 2020). Investigator-assessed response rates in *BRAF*-mutated, metastatic melanoma were 64%–75% for trametinib plus dabrafenib, cobimetinib plus vemurafenib and binimetinib plus encorafenib (Heinzerling et al., 2019). Pharmacological MEK inhibition completely abrogated tumor growth in *BRAF* mutant xenografts, whereas *RAS*-mutant tumors were only partially inhibited (Solit et al., 2006). Though drugs targeting *RAS* inhibited the RAS-RAF-MEK deregulation, wild-type *RAS* can also promote *RAS*-driven oncogenesis by downstream effectors (Muzumdar et al., 2017). This makes the treatment for *RAS*-mutant cancer more challenging. Vertical inhibition of the MAPK pathway is a promising therapeutic approach for suppressing pathway signaling and treatment resistance in *RAS*-mutant cancers (Ryan and Corcoran, 2018). Strategies to exploit combination therapies for *RAS*-mutant cancers remain promising and of great interest. SHP2 (Src homology-2 domain-containing protein tyrosine phosphatase-2) is a non-receptor protein tyrosine phosphatase and involved in downstream RAS-RAF-MEK-ERK signaling transduction (Ruess et al., 2018). Inhibition of SPH2 delayed tumor progression but not sufficient to achieve tumor regression. Inactivation of SHP2 and MEK inhibitor treatment resulted in a more sustained inhibition of the pathway, reducing resistance to inhibition of MEK (Fedele et al., 2018). Hence, the combination therapies of the new MEK inhibitor with other agents are investigated to explore the potential clinical application.

## 2 Materials and methods

### 2.1 Cell-free kinase assay

The protein kinase selectivity activity of tunlametinib was evaluated against 77 kinases. The screen study was performed by Cerep (France). Kinase inhibition activity against MEK1 was conducted in Cerep (France) and Sundia (Shanghai, China). The procedures were described in the Supplementary Materials and Methods.

### 2.2 Cell culture and proliferation assay

All cell lines were authenticated by short tandem repeat analysis and maintained following the ATCC instructions. For cell proliferation assay of tunlametinib, AZD6244 and GSK212, the study was conducted at Sundia. Anti-proliferation activity was analyzed using the 3-(4,5-dimethylthiazole-2-yl)-2,5-diphenyl-2H-tetrazolium bromide (MTT) or (3-(4,5-dimethylthiazol-2-yl)-5-(3-carboxymethoxyphenyl)-2 -(4-sulfophenyl)-2H-tetrazolium) (MTS) assay. Specific operation steps were shown in the Supplementary Materials and Methods. The combination index (CI) value between tunlametinib and other compounds was determined using CalcuSyn Version 2.1 software (Biosoft, Cambridge, UK). CI < 1, = 1 or >1 showed synergy, additive or antagonistic effects, respectively.

### 2.3 Western blot analysis

ERK and phospho-ERK levels were evaluated by Western blot analysis. Cells were seeded into 6-well plates at appropriate density (80%–90% confluence) and treated next day with the indicated inhibitors. Mice bearing A375 xenografts were treated with tunlametinib and tumor tissues were excised at 1 h after oral administration. Cells and tissues were lysed and processed as described in the Supplementary Materials and Methods.

### 2.4 Tumor cell apoptosis and cell cycle assay

Cell lines were inoculated at the density of 2×10^4^ cells per well in a 6-well plate and cultured overnight. Cells were then treated with tunlametinib, AZD6244 and GSK212 at indicated concentrations for 48 h, respectively. The cell cycle or apoptosis analysis was performed by flow cytometry. Specific operation steps were shown in the Supplementary Materials and Methods.

### 2.5 Study on pharmacokinetics of single or multiple doses administration in SD rats

SD rats were divided into 4 groups, with 3 females and 3 males in each group. Each group was administered successively: single intravenous injection of 0.5 mg/kg tunlametinib, single oral administration of 0.5, 1.5 and 4.5 mg/kg tunlametinib. Blood samples were collected pre-dosing and 0.083, 0.25, 0.5, 1, 2, 4, 8, 10 and 24 h post-dosing on day 1 and day 10. In 1.5 mg/kg oral dose group, rats were continually dosed for another 9 days. The plasma samples were analyzed by LC-MS/MS. The pharmacokinetic parameters were calculated by Phoenix ^®^WinNonlin ^®^6.3.

### 2.6 Efficacy of single drug or combination drug treatment studies *in vivo*

Tumor fragments or cell lines were implanted subcutaneously in the right flank of female BALB/c nude, NU/NU or Nod-Scid mice aged 5–8 weeks and allowed to grow to 100–300 mm^3^ on average. The specific tumor model, mice species, implanted tumor derivations are shown in Supplementary Table S1.

The efficacy of tunlametinib as monotherapy treatment was evaluated in *KRAS/BRAF* mutant or wild type xenograft model (conducted at Sundia). To compare the efficacy of MEK inhibitors tunlametinib versus MEK162, two second BRAF mutant xenograft studies were carried out (conducted at TruwayBio Suzhou). Furthermore, the efficacy of tunlametinib was tested in colorectal cancer (CRC) patient-derived model (conducted at Crown Biosciences). Tumor growth inhibition (TGI) was calculated for treatment groups using the formula: TGI% = [1-(T_i_-T_0_)/(V_i_-V_0_)]×100, T_i_ and V_i_ are the average tumor volume of the treatment group and vehicle control on the measurement day, respectively; T_0_ and V_0_ are the mean tumor volume of the treatment group and vehicle control group at the initial treatment day, respectively.

The drug combination efficacy studies were carried out in *KRAS* ^*G12C*^ mutant or *BRAF* mutant xenograft models when combined tunlametinib with SHP2/KRAS^G12C^/BRAF inhibitors, and in *KRAS* mutant xenograft model when combined with chemotherapeutic drug. Q value was applied to evaluate the synergistic effects between two drugs. Q value was calculated using the formula: Q = TGI_AB_/(TGI_A_ + TGI_B_-TGI_A_×TGI_B_), TGI_A_ or TGI_B_ represents tumor growth inhibition due to either of the two drugs respectively, and TGI_AB_ represents the growth inhibition due to the combination of the two drugs. Q > 1, = 1 or <1 indicate synergistic, additive or antagonistic effects, respectively.

The study involving animal participants were reviewed and approved by the Animal Care and Ethics Committee in Sundia MediTech Company, Ltd., Shanghai, China; TruwayBio, Suzhou, China; Crown Biosciences, Beijing, China. The *in vivo* experiment design was shown in Supplementary Figure S1. Specific operation steps were shown in the Supplementary Materials and Methods.

### 2.7 Statistical analyses

All biochemistry and cell experiments were performed in three replicates per treatment. For *in vivo* efficacy studies, data was shown as mean ± SEM. The student *t*-test was used to analyze the difference between two groups in the efficacy study. *p*-value <0.05 was considered statistically significant.

## 3 Results

### 3.1 Tunlametinib is a highly selective and potent MEK inhibitor

To determine the kinase selectivity in a panel of kinases and the inhibitory activity against MEK kinase, the cell-free enzyme assays were conducted. Tunlametinib at 10 μmol/L showed complete inhibition against MEK1 and no inhibition against other 77 kinases tested (Supplementary Information, Supplementary Table S2), suggesting a high selectivity. Further cell-free assay showed that tunlametinib had a significant inhibitory activity against target kinase MEK1, and the IC_50_ was 1.9 nM (Figure 1B). In addition, under the comparison study, tunlametinib exhibited approximately 19-fold greater inhibitory activity against MEK1/MAP2K1(h) kinase than the lead compound MEK162, with IC_50_ value of 12.1 ± 1.5 nM *versus* 223.7 ± 16.9 nM (Figure 1C).

### 3.2 Cell proliferation assay

In a panel of cell lines with *RAS* or *RAF* mutation, tunlametinib dramatically inhibited cell proliferation, with IC_50_ values ranging between 0.67 and 59.89 nmol/L. In contrast, tunlametinib, at concentrations up to 10 μmol/L, had minimal inhibitory effect on the proliferation of *RAS/RAF* wild-type tumor cells (H1975) and normal cells (MRC-5). Furthermore, the head-to-head comparison results showed that the inhibitory activity of tunlametinib was similar to GSK212, and more potent than that of AZD6244 (10–100 times). The IC_50_ values and the proliferation inhibition curves are presented in Table 1 and Supplementary Information (Supplementary Figure S2). These results demonstrated that tunlametinib is more effective for *RAS/RAF*-mutant cell lines with improved potency compared to AZD6244.

**TABLE 1:** IC_50_ values of a panel of cancer cell lines exposed to tunlametinib, AZD6244 and GSK212.^a^

Cell lines	Tumor type	Mutation status	Tunlametinib IC_50_ ± SEM (nM)	AZD6244 IC_50_ ± SEM (nM)	GSK212 IC_50_ ± SEM (nM)
A375	Melanoma	BRAF^V600E^	0.86 ± 0.07	67.52 ± 0.07	0.74 ± 0.01
COLO 205	Colon	BRAF^V600E^	0.94 ± 0.26	54.33 ± 2.21	0.74 ± 0.02
Colo-829	Melanoma	BRAF^V600E^	3.46 ± 0.27	301.10 ± 88.79	1.78 ± 0.00
HT-29	Colon	BRAF^V600E^	2.35 ± 0.03	175.28 ± 19.26	1.45 ± 0.20
Calu-6	Lung	KRAS^Q61K^	10.07 ± 1.18	2305.07 ± 203.56	10.56 ± 0.07
A549	Lung	KRAS	59.89 ± 11.06	5732.45 ± 1028.89	45.35 ± 4.76
HL-60	Myeloma	RAS	0.67 ± 0.28	35.26 ± 20.92	0.60 ± 0.28
H1975	Lung	BRAF^WT^, KRAS^WT^	>1000	>50,000	>1000
MRC-5	Lung	Normal cell	>1000	>50,000	>1000

^a^ *In vitro* cell viability was determined by MTS, assay.

### 3.3 Inhibition of ERK phosphorylation in cultured cells

**FIGURE 2:**
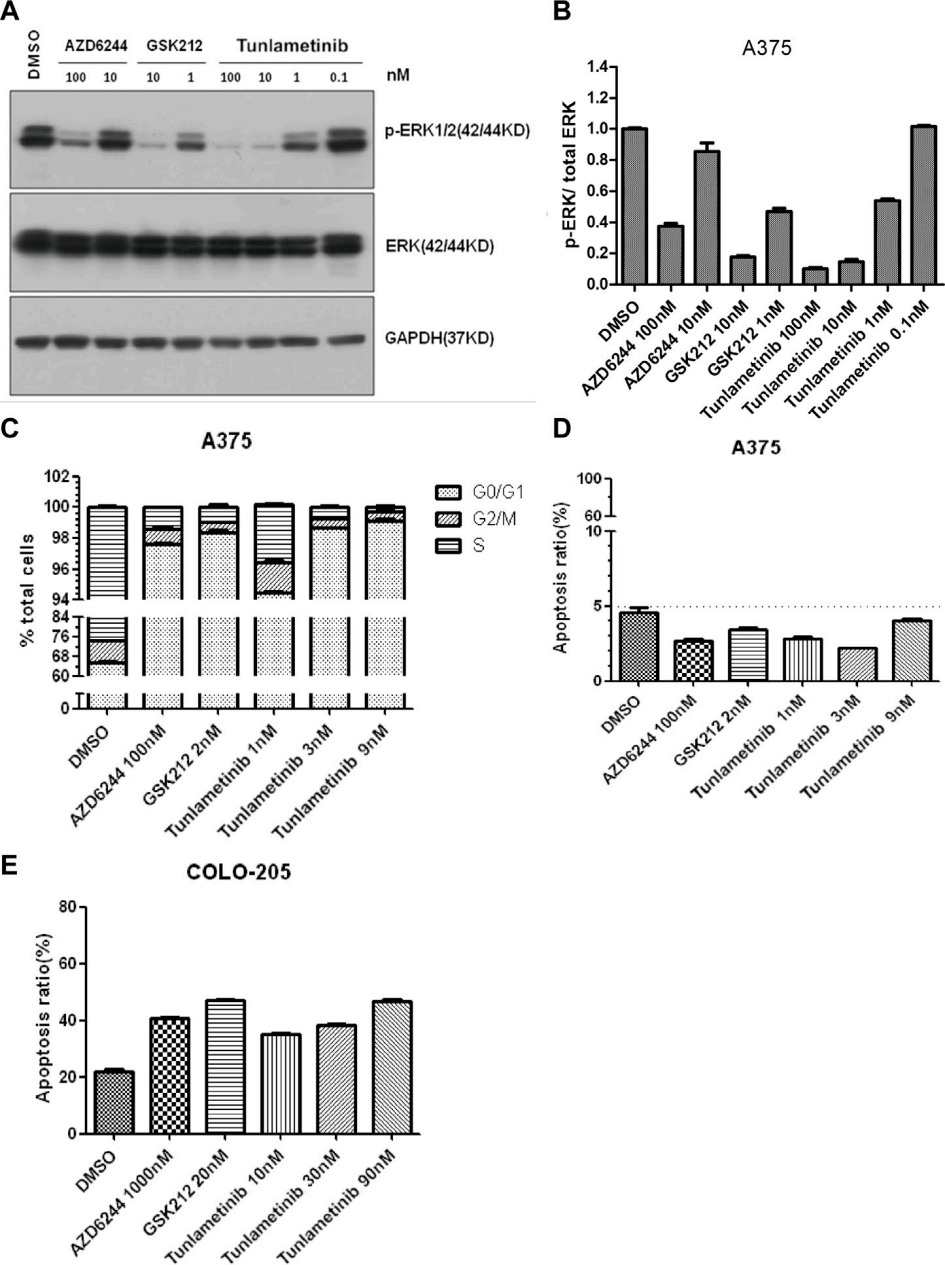
The effect of tunlametinib on p-ERK/total ERK inhibition, cell cycle and apoptosis. **(A,B)** The level of p-ERK/total ERK in A375 cells after treated with different concentrations of compounds. A375 cells were treated with tunlametinib at 0.1, 1, 10, 100 nM, with AZD6244 at 10, 100 nM, and with GSK212 at 1, 10 nM. **(C,D)** A375 cells were treated with tunlametinib at 1, 3, 9 nM, with AZD6244 at 100 nM, and GSK212 at 2 nM. **(E)** COLO 205 cells were treated with tunlametinib at 10, 30, 90 nM, with AZD6244 at 1000 nM, and GSK212 at 20 nM.

The ability of tunlametinib to inhibit MEK1/2 kinase activity in their cellular environment was evaluated by measuring the phosphorylation state of ERK1/2, the direct substrates of MEK1/2. Furthermore, the inhibitory ability of tunlametinib was compared with another two MEK inhibitors GSK212 and AZD6244. Western blot assays were introduced to detect pERK level after 48-h inhibitors treatment. Results (Figures 2A, B) showed that the ERK phosphorylation level of *BRAF*-mutated melanoma A375 cells decreased in a dose-dependent manner and reached almost completely blocked under treatment of tunlametinib at 100 nM. Besides, the IC_50_ value of tunlametinib on inhibition of ERK phosphorylation in A375 cells was close to the anti-proliferation IC_50_ value *in vitro*, which was about 1.16 nM. Compared with the other two inhibitors, the inhibitory effect of tunlametinib at 1 nM and 10 nM was similar to that of GSK212, but much better than that of AZD6244, consistent with the reduced proliferation of cells.

### 3.4 Cell cycle and apoptosis

A375 cells were treated with tunlametinib, AZD6244 and GSK212 for 48 h to evaluate the effect on cell cycle. Flow cytometry analysis showed that tunlametinib could dose-dependently increase the proportion of G0/G1 phase in A375 cells at concentration from 1 nM to 9 nM (Figure 2C). Additionally, its effect on cell cycle arrest was more potent than that of AZD6244, and similar to GSK212.

A375 and BRAF-mutated colon cancer COLO 205 cells were treated with tunlametinib, AZD6244, and GSK212 for 48 h to evaluate the effect on cell apoptosis. The proportion of apoptosis cells after compound treatment were within normal limits, suggesting no significant apoptosis-inducing effect on A375 cells (Figure 2D). Meanwhile, the percentage of apoptotic cells in control group was 21.9% on COLO 205 cells. After treated with 1 μM AZD6244 or 20 nM GSK212, the apoptosis rate was 40.6% and 47.0%, respectively; while the proportion of apoptosis was 35.1%, 38.4% and 46.6% after treated with 10, 30 and 90 nM of tunlametinib (Figure 2E). In summary, tunlametinib could dose-dependently induce apoptosis COLO 205 cells, and its effect was stronger than that of AZD6244, but slightly weaker than GSK212.

### 3.5 Pharmacokinetics profile

After oral administration of 0.5, 1.5 and 4.5 mg/kg tunlametinib to Sprague-Dawley (SD) rats, the time to reach peak drug concentration (T_max_) was around 0.5–4 h. The area under curve from 0 to 24 h (AUC_0–24h_) were 3564.8 ± 711.8 ng/mL*h, 6658.2 ± 2126.7 ng/mL*h and 21,581.2 ± 9058.4 ng/mL*h, respectively, demonstrating a good dose-proportionality. The mean absolute bioavailability was 86.6% ± 22.6%, 53.0% ± 15.8% and 57.6% ± 22.5%, respectively, showing a favorable oral bioavailability. There was no significant difference of PK parameters between single dose and 10-day repeated doses, indicating no drug exposure accumulation.

### 3.6 Anti-tumor activities of tunlametinib as monotherapy *in vivo*

**FIGURE 3:**
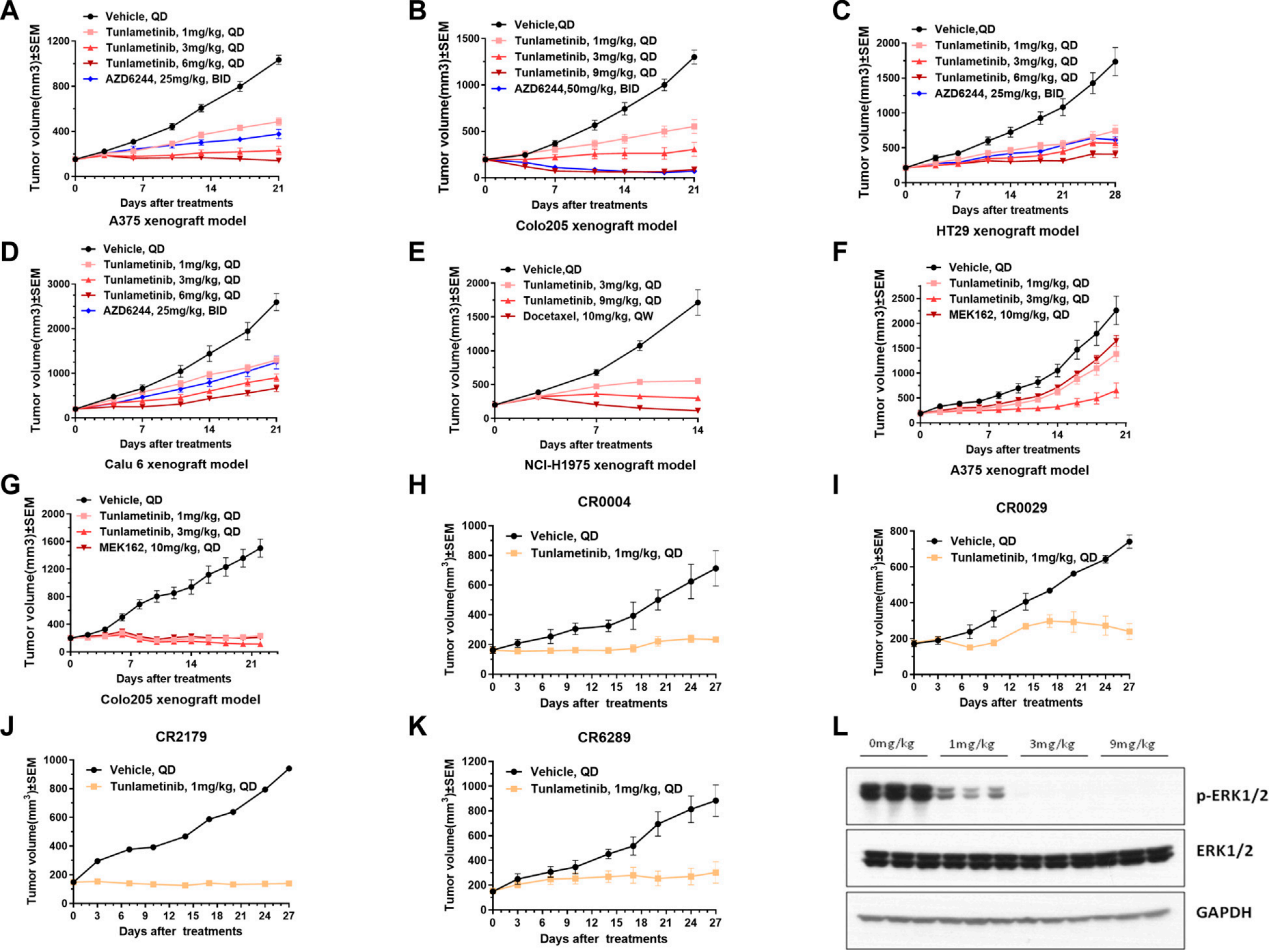
Tumor growth of cell line-derived xenografts (CDXs) or patient derived CRC xenograft (PDX) under single agent treatment and p-ERK protein levels in A375 tumor tissue. **(A,C,D)** A375, HT-29, Calu-6 CDX_S_ were treated with vehicle, tunlametinib (1, 3, 6 mg/kg, QD) or AZD6244 (25 mg/kg, BID). **(B)** COLO 205 CDX was treated with vehicle, tunlametinib (1, 3, 9 mg/kg, QD) or AZD6244 (50 mg/kg, BID). **(E)** NCI-H1975 CDX was treated with vehicle, tunlametinib (3, 9 mg/kg, QD) or docetaxel (10 mg/kg, QW). **(F,G)** A375, COLO 205 CDXs were treated with vehicle, tunlametinib (1, 3 mg/kg) or MEK162 (10 mg/kg, QD). **(H–K)** CR0004, CR0029, CR2179, CR6289 PDXs were treated with vehicle and tunlametinib (1 mg/kg). **(L)** A375 CDX was treated with tunlametinib (0, 1, 3, 6 mg/kg, PO) and tumor tissues were excised at 1 h after single oral dosing of tunlametinib. For A375 CDX model, N = 14 mice in vehicle group, and N = 7 in tunlametinib and AZD6244 treatment groups. For other CDX models, N = 8 mice in each group. For PDX model, N = 2 mice in vehicle group, and N = 3 mice in tunlametinib treatment groups. Data was shown as mean ± SEM, ns *p* > 0.05, * *p* < 0.05, ** *p* < 0.01, ****p < 0.0001, student *t*-test.

The anti-tumor activity of tunlametinib as monotherapy was investigated in *BRAF/KRAS* mutant or wild type xenograft model. Consistent with the findings *in vitro*, tunlametinib inhibited tumor growth in a dose-dependent manner in all the xenograft models. Tunlametinib at the low dose exhibited stronger inhibition of the *BRAF* and *KRAS* mutant xenograft model compared to AZD6244 at high dose (Figures 3A–D). Tunlametinib also demonstrated stronger inhibition of *BRAF*-mutant melanoma and colorectal xenograft when compared to MEK162 (Figures 3F, G). Moreover, tunlametinib also showed potent anti-tumor effect in the *BRAF/KRAS* wild type xenograft model mice (Figure 3E). In four patient-derived xenograft (PDX) CRC models harboring *BRAF* mutation (CR0004, CR0029, CR2179, CR6289), tunlametinib at dose of 1 mg/kg QD, responded (>79% TGI) with all the models exhibiting significant tumor growth suppression (*p* < 0.05) (Figures 3H–K). No significant change in body weight of vehicle and treatment group in all xenograft models (Supplementary Figure S3).

### 3.7 Tunlametinib resulted in a profound inhibition of ERK phosphorylation in tumor tissues

Tunlametinib was mechanistically evaluated in mice for antitumor activity against A375 xenografts. ERK phosphorylation was analyzed as the biomarker of MAPK pathway inhibition since ERK is the direct substrate of MEK. Tumor tissues of mice were collected at 1 h after orally administration of tunlametinib with 1 mg/kg, 3 mg/kg or 9 mg/kg. As shown in Figure 3L, the phosphorylation of ERK in tumor tissue was significantly inhibited at 1 mg/kg and achieved completely inhibition at 9 mg/kg. The inhibition percentage of p-ERK were 80.37%, 99.99% and 100.00% respectively, indicating that tunlametinib could inhibit ERK phosphorylation *in vivo* in a dose-dependent manner.

### 3.8 The synergistic effects of tunlametinib and BRAF/KRAS^G12C^/SHP2 inhibitors or chemotherapeutic agent on cancer cell growth

Increasing evidence showed that multi-targeted combination therapy delayed the onset of acquired resistance, leading to increased progress-free survival and overall survival (Broman et al., 2019). For *BRAF* mutant colon cancer and melanoma, monotherapy showed limited efficacy, however combination therapy confers a promising therapy approach. Tunlametinib combined with Vemurafenib (a BRAF inhibitor) showed synergistic effect on colorectal COLO 205 and HT-29 under the inhibition extent from 9% to 69% and 21%–81%, respectively. In addition, the minimum of CI value occurred under the IC_50_ treatment of both inhibitors. A similar results were observed on melanoma A375, with only two concentration groups showed antagonism.

**FIGURE 4:**
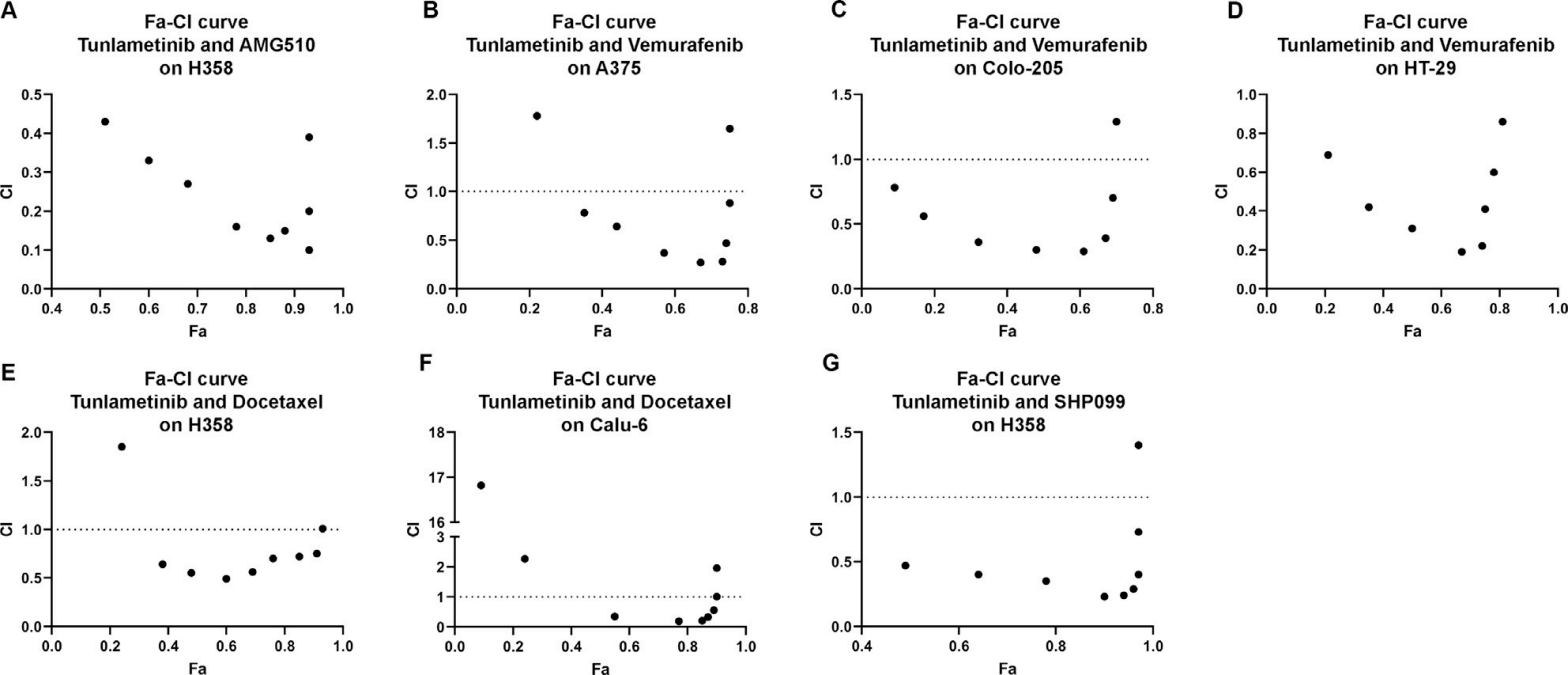
Synergistic effects of tunlametinib combined with **(A)**, KRAS^G12C^ inhibitor, **(B–D)**, BRAF inhibitor, **(G)** SHP2 inhibitor or **(E,F)**, chemotherapeutic agent on BRAF- or KRAS-mutant cell lines. Fa-CI curves of KARS- or BRAF- mutant cell lines treated with tunlametinib combined with AMG510, vemurafenib, docetaxel, or SHP099. CI = 1 line represents additive effect; Dots below CI = 1 line represent synergistic effect; Dots above CI = 1 line represent antagonistic effect.

It has been proven that the inhibition of phosphorylated ERK1/2 level in tumor cells with *KRAS* mutations may cause wide-type RAF phosphorylation and further result in activation of MEK, thereby restoring pERK level quickly and acquiring rebound. Therefore, it is necessary to introduce MEK inhibitor and KRAS inhibitor combination therapies. The fraction affected-CI curve of tunlametinib and AMG 510 (a KRAS^G12C^ inhibitor) effects on NSCLC H358 indicated that synergistic effect almost existed in all concentrations tested. Notably, CI value decreased to 0.1, which indicated an intense synergistic effect (Figure 4).

Docetaxel, as a chemotherapeutic agent, is a commonly used microtubule-stabilizing cytotoxic drug that has a great potential in clinical application mainly on NSCLC treatment. Combination of tunlametinib with docetaxel in lung cancer H358 and Calu-6 cells showed synergistic effects as the CI value was below 1.

SHP2 is a non-receptor protein tyrosine phosphatase encoded by the PTPN11 gene and is involved in cell growth and differentiation via the MAPK signaling pathway. SPH2/MEK inhibitor combinations prevent adaptive resistance in *KRAS* mutant cancer model (Fedele et al., 2018). SHP2 inhibitor SHP099 combined with tunlametinib was used to determine whether they have synergistic effect. As expected, synergism was observed on H358 under the inhibition extent from 48% to 97%.

### 3.9 Combination therapy for cancers with tunlametinib and SHP2/KRAS^G12C^/BRAF inhibitors or chemotherapeutic agent using animal models

**FIGURE 5:**
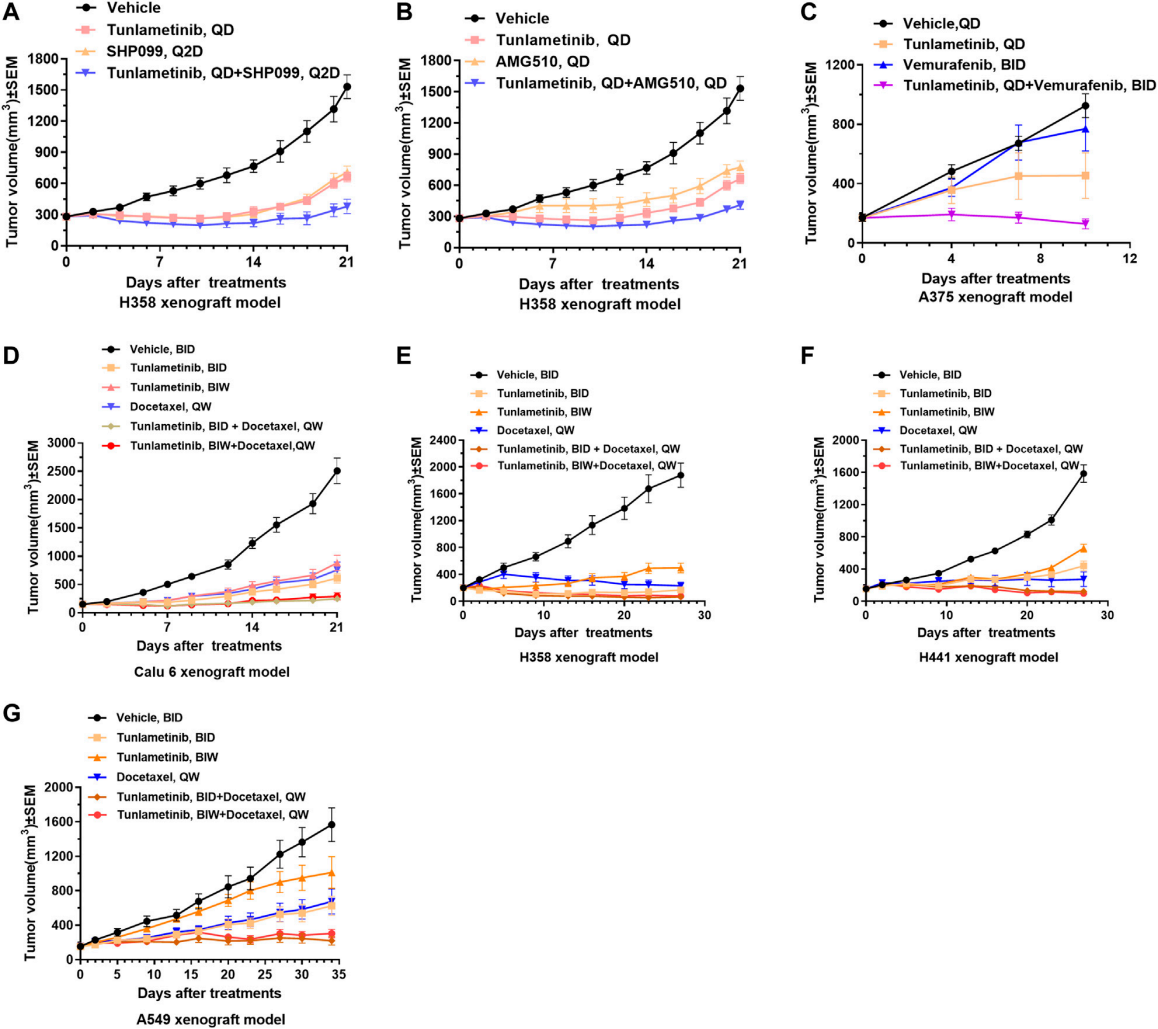
Tumor growth curves of combination treatments in cell line-derived xenografts. Tunlametinib combined with SHP2/KRAS^G12C^/BRAF inhibitors in *KRAS* or *BRAF* mutant xenografts. **(A,B)** Tunlametinib (0.25 mg/kg, then decreased to 0.125 mg/kg from day 10, QD, P.O.) combined with SHP099 (50 mg/kg, then decreased to 25 mg/kg from day 10, Q2D, P.O.) or AMG510 (3 mg/kg, QD, P.O.) in H358 xenograft model (n = 8 mice per group). **(C)** Tunlametinib (1 mg/kg, QD, P.O.) in combination with vemurafenib (25 mg/kg, BID, P.O.) in A375 xenograft model (n = 6 mice per group). **(D)** Tunlametinib (0.5 mg/kg, BID or 3.5 mg/kg, BIW, P.O.) combined with docetaxel (10 mg/kg, then decreased to 7.5 mg/kg from day 8, QW, i. v.) in Calu-6 xenograft models (n = 8 per group). **(E)** Tunlametinib (0.5 mg/kg BID or 3.5 mg/kg BIW, P.O.) combined with docetaxel (10 mg/kg for 2 weeks, decreased to 7.5 mg/kg for 1 week, and 5 mg/kg for 1 week, QW, i. v.) in H358 xenograft models (n = 8 per group). **(F)** Tunlametinib (0.5 mg/kg BID or 3.5 mg/kg BIW, P.O.) combined with docetaxel (10 mg/kg for 2 weeks, then decreased to 7.5 mg/kg for 2 weeks, QW, i. v.) in H441 xenograft models (n = 8 per group). **(G)** Tunlametinib (0.5 mg/kg BID or 3.5 mg/kg BIW, P.O.) combined with docetaxel (10 mg/kg for 1 week, then decreased to 7.5 mg/kg for 4 weeks, QW, i. v.) in A549 xenograft models (n = 8 per group). Data were shown as mean ± SEM, *p < 0.05, **p < 0.01, ***p < 0.001, student *t-*test.

The combination of tunlametinib with SPH2 inhibitor SHP099 enhanced the anti-tumor effect and resulted in synergistic inhibition of *KRAS* ^*G12C*^ mutant tumors (Q = 1.02, *p* < 0.01) (Figure 5A). Likewise, tunlametinib (1 mg/kg, QD, P.O.) combined with KRAS^G12C^ inhibitor AMG 510 resulted in stronger efficacy and exhibited a synergistic effect in *KRAS* ^*G12C*^ mutant xenograft model (Q = 1.02, *p* < 0.01) (Figure 5B), consistent with *in vitro* result. Moreover, combination treatment of tunlametinib with vemurafenib resulted in remarkable tumor inhibition in *BRAF* mutant melanoma cells, which indicates a strong synergistic effect (Q = 1.51, *p* < 0.05) (Figure 5C). Tunlametinib in two oral dosing schedules combined with docetaxel synergistically in all four *RAS* mutant xenograft (Q > 1, *p* < 0.05) (Figures 5D–G). During the treatment period, no significant changes in body weight were observed in all xenograft models (Supplementary Figure S4).

## 4 Discussion

Here we describe the preclinical characterization of tunlametinib, a novel, highly selective, potent, and orally available small molecule MEK inhibitor. Tunlametinib showed remarkable efficacy against *RAS/RAF* mutant cancers *in vitro* and *in vivo*. This indicates that tunlametinib could provide an effective therapy approach for *RAS/RAF* mutant cancers. Tunlametinib emerged as the top candidate from an optimization of molecular structure, as this compound incorporated both improved potency and favorable PK properties. Trametinib, as the first approved MEK inhibitor, has blood drug accumulation with the mean accumulation ratio (day15/day1) with repeat dose of 2 mg approximate 6.0 (Food and Drug Administration, 2013), whereas tunlametinib showed minimal drug accumulation in preclinical and clinical studies (Zhao et al., 2022). Compared with other three FDA-approved MEK inhibitors, tunlametinib showed much more potent both *in vitro* and *in vivo*. The phosphorylation of ERK, biomarker of MEK inhibition, was evidently inhibited after tunlametinib was administered to cells *in vitro* or xenograft model *in vivo*. Moreover, the extent of this inhibition was in dose-dependent manner and maximally approached to 100%. This suggests that tunlametinib efficiently inhibits the MAPK pathway and results in cell growth suppression. Like GSK212 and AZD6244, tunlametinib induced A375 cell cycle arrest at G0/G1 phase, but did not result in apoptosis, indicating that A375 cells probably respond to tunlametinib by inhibition of cell proliferation through cell cycle or other mechanisms, rather than apoptosis. However, tunlametinib induced apoptosis in COLO 205 cells. This difference could be explained by the possibility MEK inhibitor may induced apoptosis by differentiated mechanism, depending on cell types (Davies et al., 2007). Collectively, tunlametinib demonstrated favorable pharmacokinetic profile and good drug properties.

The challenges in the treatment of some subtype of *RAS* mutant cancers and acquired drug-resistance due to an abundance of escape mechanisms present highlight the need of new strategies to improve clinical outcomes (Heppt et al., 2015). This study further proved that tunlametinib synergistically enhances the potency and efficacy of BRAF inhibitors, SHP2 inhibitors, KRAS^G12C^ inhibitors and chemotherapy agent docetaxel. Tunlametinib may potentially improve therapeutic responses and reduce the likelihood of acquired resistance in cancer patients, especially in *RAS* mutant cancers. *NRAS* ^*Q61K*^ mutant neuroblastomas were distinctly resistant to SHP2 inhibitors; however, combinations of SHP2 inhibitor with MEK inhibitor were synergistic and reversed resistance to SHP2 inhibition (Valencia-Sama et al., 2020). RTK-driven feedback activation widely exists in *KRAS*-mutant cancer cells, and this pathway feedback activation is mediated through mutant *KRAS*, at least for the G12C, G12D, G12V variants (Lu et al., 2019). Combining SHP2 and MAPK pathway inhibitor for treating *KRAS*-mutant cancers is a rationale strategy in the clinic. Our study demonstrated that the MEK inhibitor tunlametinib combining SPH2 inhibitor SHP099 showed a synergistic effect of tumor inhibition, indicating a promising potential in the clinical utility. Research showed that *KRAS*-amplified cancers are insensitive to MAPK blockade due to adaptive response by rapidly increasing KRAS-GTP levels. However, inhibition of SPH2 could enhance the sensitivity of KRAS-amplified cancer model to MEK inhibition (Wong et al., 2018). Mechanistically, SHP2 inhibitors suppressed activation of *KRAS* mutants, impeded SOS/RAS/MEK/ERK1/2 reactivation in response to MEK inhibitors (Fedele et al., 2018). It is possible that combination of tunlametinib with SPH2 inhibitor could potentially confer a beneficial outcome for wide-type *KRAS*-amplified cancers and could have therapeutic utility in multiple cancers.

In this study, combining tunlametinib with vemurafenib demonstrated a remarkable synergistic effect of tumor inhibition, indicating a sustainable inhibition of MAPK signaling and likely overcoming paradoxical MAPK activation. The novel MEK inhibitor tunlametinib in combination with KRAS^G12C^ inhibitor AMG510 demonstrated high activity in preclinical xenograft models of *KRAS*-mutant cancers, rendering a potentially effective treatment for *RAS*-mutant cancers in clinic. This study could support the combination therapies of tunlametinib in clinical.

A retrospective multicenter analysis of 364 patients concluded that additional MEK inhibition to immune checkpoint inhibition has potential to increase survival of *NRAS*-mutated melanoma patients and improve clinical benefit (Kirchberger et al., 2018). Patients with prior immunotherapy with anti- Cytotoxic T lymphocyte-associated antigen (CTLA-4) or anti-PD-1 antibody may even experience more durable responses to MEK inhibitor. Combining MEK inhibitor and anti-PD-ligand 1 (PD-L1) markedly enhances tumor response in mice with KRAS-mutated colon cancer xenografts. MEK inhibition increased the number of intratumoral antigen-specific CD8^+^ effector T cell. Tunlametinib as a novel MEK inhibitor with improved efficacy might be an ideal combination partner with immune checkpoint inhibitors.

Overall, our findings constitute a preclinical data of tunlametinib and offer a precision medicine option available that could be tailored to individual mutations and cancers. These data supported the progression of tunlametinib into a first-in-human clinical study (Zhao et al., 2022; Wang et al., 2023). Currently, the first-in-human phase 1 (ClinicalTrials.gov number, NCT03973151, NCT04683354) and pivotal clinical study of tunlametinib (NCT05217303) as monotherapy, and the phase 1 study as combination therapy (NCT05263453) have been completed. More pivotal trials are ongoing (NCT03781219, NCT05233332).

## Data Availability

The original contributions presented in the study are included in the article/Supplementary Material, further inquiries can be directed to the corresponding author.

## Abbreviations

CI, combination index; CRC, colorectal cancer; CTLA-4, Cytotoxic T lymphocyte-associated antigen; ERK, extracellular regulated protein kinases; FDA, U.S. Food and Drug Administration; MAPK, Mitogen-activated protein kinase; MEK, mitogen-activated protein kinase kinase; MTS, 3-(4,5-dimethylthiazol-2-yl)-5-(3-carboxymethoxyphenyl)-2-(4-sulfophenyl)-2H- tetrazolium); MTT 3, (4,5-dimethylthiazole-2-yl)-2,5-diphenyl-2H-tetrazolium bromide; NSCLC, non-small cell lung cancer; PDX, patient-derived xenograft; PD-L1, PD-ligand 1; PK, pharmacokinetics; SHP2, Src homology-2 domain-containing protein tyrosine phosphatase-2; TGI, tumor growth inhibition; T_max_, time to reach peak drug concentration.
