# Supplementary material for: Preclinical characterization of tunlametinib, a novel, potent, and selective MEK inhibitor
Source: Front Pharmacol. 2023 Sep 21;14:1271268. doi: 10.3389/fphar.2023.1271268 (PMC10557067; doi:10.3389/fphar.2023.1271268)

**Supplementary Data**

**Title: Preclinical Characterization of Tunlametinib, a Novel, Potent, and Selective MEK Inhibitor**

**Running Title: Tunlametinib, a potent and selective MEK inhibitor**

**Yahong Liu ^#^, Ying Cheng ^#^, Gongchao Huang, Xiangying Xia, Xingkai Wang, and Hongqi Tian***

**Authors' affiliations:** Shanghai Kechow Pharma, Inc., Shanghai 201203, China.

**Keywords:** MEK inhibitor; RAF/RAS mutant cancer; drug combination; high potency; preclinical study

***Corresponding author:** Hongqi Tian, Address: No.780 Cailun Road, Zhangjiang Hi-Tech Park, Pudong New Area, Shanghai, China. ZIP Code: 201203; Phone: +86-21-68750976; Fax: 021-68750976; Email: [tianhq@kechowpharma.com](mailto:tianhq@kechowpharma.com).

# These authors contributed equally.

**Supplementary Materials, Methods, Tables and Figures**

**Kinase selectivity assay**

This assay was conducted by Cerep Inc. Briefly, tunlametinib was added to the 384-well plate at a final concentration of 10 μM. The kinase tested (approximately 0.4 ng, Cerep) were added respectively, and substrate at indicated concentration and 10 μM of ATP were added to initiate the reaction. After incubation at room temperature for 60 minutes, EDTA was added to terminate the reaction. After 5 minutes termination, the detection buffer was added and incubated for 60 minutes at room temperature. Signal was readout by EnVision HTS microplate reader (Model: 2104-0010, Perkin Elmer). Inhibition rates (%) of tunlametinib against kinases were calculated using the formula: 100*(1-(measured kinase activity/control kinase activity)).

**Inhibition activity against MEK1 kinase assay**

Tunlametinib was mixed with the MEK1 enzyme (4.92 ng) in a buffer containing 40 mM Hepes/Tris (pH 7.4), 0.8 mM EGTA/Tris, 8 mM MgCl_2_, 3.6 mM DTT and 0.008% Tween 20. Thereafter, the reaction is initiated by adding 50 nM of inactive ERK2 and 30 µM ATP, and the mixture is incubated for 60 min at room temperature. For control basal measurements, the enzyme is omitted from the reaction mixture. Following incubation, the reaction is stopped by adding 13 mM EDTA. After 5 min, Ulight-anti-GST label and anti-phopho-p44/42MAPK antibody labeled with europium chelate are added. After 60 more min, the fluorescence transfer is measured at λex=337 nm, λem=620 nm and λem=665 nm using a microplate reader (Envision, Perkin Elmer). The enzyme activity is determined by dividing the signal measured at 665 nm by that measured at 620 nm (ratio). The results are expressed as a percent inhibition of the control enzyme activity.

The reaction for comparing tunlametinib to MEK162 on the inhibition of MEK1 was carried out in a 384-well plate with an incubation mixture composed of 1× kinase buffer (containing 50 mM HEPES (pH 7.5), 0.015% BRIJ-35, 0.01% TritonX-100), 0.025 nM MEK1 (Carna, 07-141, 12CBS-0362D), 40 nM inactive ERK2 (Carna, 04-143-10, 12CBS-0130E). Tunlametinib and MEK162 (Aichonchem, 606143-89-9) were gradient diluted at a ratio of 1:3 and added to the final mixture to make series of concentrations as indicated (containing 1% DMSO; triplicate for each concentration). After 60 minutes pre-incubation at room temperature, the reactions were initiated by adding 10 μM ATP and kinase substrate8 (GL, 112396, P171207-MJ112396) and incubated at room temperature for 60 minutes. After the reactions were terminated by stop solution. The signals were measured using Caliper EZ Reader and the data were analyzed.

**Cell lines and cell proliferation assay**

For cell proliferation assay of tunlametinib, AZD6244 and GSK212, the study was conducted at Sundia. A375, HT-29 and Calu-6 cell lines were purchased from the typical culture preservation commission cell bank of the Chinese Academy of Sciences in China. COLO 205 cell line was from Institute of Basic Medical Sciences, Chinese Academy of Medical Sciences in China. H358 cell line was obtained from EK-bioscience. Cells were seeded into 96-well plates at an appropriate density, and 24 h later, diluted drug in different concentrations was added to each test well in triplicate. The cell viability was tested 5 days (H358 was analyzed for 7 days) after the cells were plated according to the procedure originally described by Mosmann [1]. Survival rate was calculated using the formula: Survival rate = (Mean absorbance of experimental wells-Mean absorbance of DMSO wells) / (Mean absorbance of control wells-Mean absorbance of DMSO wells) × 100%. IC_50_ was determined from the regression of a plot of the logarithm of the concentration versus survival rate by GraphPad Prism 6 using the non-linear regression Dose–Response Inhibition (four parameters) Model.

**Western blot analysis**

A375 were inoculated in 6-well plates with 3×10^5^ cells per well and cultured overnight. AZD6244 (final concentration 100, 10 nM), GSK212 (final concentration 10, 1 nM) and tunlametinib (final concentration 100, 10, 1, 0.1 nM) were added after 3 h serum-starvation. After 48 hours treatment, the cells were washed twice with pre-cooled PBS, 100 μL of 1×SDS (L5750, Sigma) lysis buffer containing phosphatase inhibitor and protease inhibitor was added, and the plates were placed on ice for 20 min, then cell lysate were centrifuged at 13000 rpm at 4°C for 15 minutes. Supernatant was collected and protein concentrations were determined by BCA method (PICPI23227, Thermo Scientific). Samples containing an equal amount of protein (20 μg) were mixed with 4×LDS (NP0008, Invitrogen) for a total volume of 50 μL (containing 10% sample reducing agent (NP0009, Invitrogen), then heated at 95 ℃ for 8 min, and centrifuged at 13000 rpm for 3 min in a 4 ℃. Equal amounts of samples were resolved on NuPAGE® Novex 4-12% Bis-Tris Gel (NP0323BOX, Invitrogen), and then were transferred to PVDF membrane (75696G, Pall), ERK1/2 protein (both phosphorylated and total) was blotted with the indicated antibodies diluted at a 1:1000 or 1:2000 ratio (anti-p-ERK1/2: CST, 4370S; anti-ERK1/2: CST, 4695). The internal reference was detected by horseradish peroxidase-conjugated anti-GAPDH antibody diluted at 1:10000 ratio (KC-5G5, KangChen Bio-tech). The chemiluminescent signal was generated with ECL (PerkinElmer, NEL104001EA) and recorded with photographic film.

**Tumor cell apoptosis and cell cycle assay**

Tumor cell lines were inoculated in 6-well plates at a density of 2×10^4^ cells/well. Tunlametinib, AZD6244 and GSK212 were added, respectively, and vehicle control group was treated with culture medium containing 0.5% DMSO. After 48 hours treatment, apoptosis and cell death were measured by staining with annexinV-FITC and prodidium iodide (556547, BD Biosciences). After fixation by 70% ethanol, cell cycle was detected by PI/RNase Staining Buffer (BD Pharmingen, 550825). Both apoptosis and cell cycle were measured by flow cytometry (Accuri C6, BD biosciences).

**Animal housing**

Before tumor inoculation, approximately one week were allowed for animal to get accustomed to the laboratory environment, all the mice were maintained in a pathogen-free environment. All cages, food and water were sterilized before use. Mice were housed in an environmentally controlled room (temperature at a range of 20~26℃, humidity at 40~70%) on a 12-hour light/dark cycle.

**Cell-derived xenograft (CDX) and patient-derived xenograft (PDX) models**

When implanted with tumor fragment, tumor bearing mice were given euthanasia and then tumors were excised, washed with saline, cut into 1 mm^3^ tumor fragments and implanted subcutaneously at the right flank of each mouse for tumor development. While injected with cell lines, 5×10^6^ cells in a volume of 0.1 mL (PBS: matrigel=1:1 or PBS only) or 2.5×10^6^ cells in 0.15 mL RPMI 1640 (Gibco, C11875500BT) were injected subcutaneously on the right flank of each mouse. For primary human colorectal cancer model (CR0004, CR0029, CR2179 and CR6289), mice were inoculated subcutaneously at the right flank with fragment (2-3 mm in diameter) for tumor development.

**Efficacy of single agent treatment studies *in vivo***

The efficacy of tunlametinib as single drug treatment was evaluated in A375, COLO 205, HT-29, Calu-6 and NCI-H1975 xenograft models. Tumor fragments or cell lines were implanted subcutaneously in the right flank of BALB/c nude, NU/NU or Nod-Scid mice and allowed to grow to 100 to 300 mm^3^ on average. Vehicle control group were treated with 10 mL/kg vehicle, P.O., QD (0.5% Hydroxy propyl methyl cellulose / 0.2% Tween 80) (HPMC, PD323924; Tween 80, 20130528, Sundia). For A375, COLO 205, HT-29 and Calu-6 xenografts, the positive control group were administered to AZD6244 (25 mg/kg or 50 mg/kg, P.O., BID) solubilized in 20% SBE-β-CD (73747519, Sundia). The treatment groups were dosed tunlametinib (1, 3, 6 or 9 mg/kg, P.O., QD) which was solubilized in vehicle solution. For NCI-H1975 xenograft model, the reference drug was docetaxel at 10 mg/kg solubilized in 5% ethanol and 5% Tween 80 in saline solution once a week by i.v. injection. The dosages of tunlametinib were set at 3 mg/kg and 9 mg/kg, QD.

To evaluate efficacy of MEK inhibitors tunlametinib versus MEK162, two second A375 and COLO 205 xenograft studies were carried out. MEK162 at 10 mg/kg (1% CMC / 0.5% Tween 80) (CMC, TruwayBio Suzhou) was the positive control group. The treatment groups received tunlametinib (1 mg/kg and 3 mg/kg, PO, QD) which was solubilized in vehicle solution. For BRAF mutant colorectal cancer PDX models, the vehicle was formulated according to the following ratio (PEG400:Kolliphor® RH 40: PBS (v:v:v=20:20:55)):100%DMSO (v:v=19:1). The treatment group received tunlametinib at 1 mg/kg dissolved in 20% SBE-β-CD via oral gavage once daily. Tumor growth inhibition (TGI) was calculated for treatment groups using the formula: TGI% = [1-(T_i_-T_0_)/(V_i_-V_0_)]×100, T_i_ and V_i_ are the average tumor volume of the treatment group and vehicle control on the measurement day, respectively; T_0_ and V_0_ are the mean tumor volume of the treatment group and vehicle control group at the initial treatment day, respectively.

**Efficacy of combination drug treatment studies *in vivo***

When combined with targeted therapy agents, the studies were carried out in H358 (KRAS^G12C^) and A375 (BRAF^V600E^) xenograft model. The vehicle control groups were treated with 10 mL/kg or 5 mL/kg vehicle, QD, P.O., respectively. In H358 xenograft model, the treatment groups were given tunlametinib (0.25 mg/kg, then decreased to 0.125 mg/kg from day 10, QD, P.O.) / AMG510 (3 mg/kg, PO, QD, 0.5% MC) or SHP099 (50 mg/kg, and decreased to 25 mg/kg from Day 10, Q2D, P.O., 0.6% MC / 0.5% Tween 80 in 0.9% saline) / combination (MC, C1506015, Suzhou TruwayBio). For A375 xenograft model, the treatment group received tunlametinib (1 mg/kg, QD, P.O.) / vemurafenib (25 mg/kg, BID, P.O., 2% Klucel LF) / combination (Klucel LF, 53300, Shanghai chineway).

When combined with chemotherapeutic drug, the vehicle control group was treated with 10 mL/kg vehicle, BID, P.O.. For Calu-6 xenograft model, tunlametinib single drug treatment groups were treated with tunlametinib (0.5 mg/kg BID or 3.5 mg/kg BIW, P.O.). The ancillary drug group were treated with docetaxel (10 mg/kg, then decreased to 7.5 mg/kg from day 8, QW, dissolved in 2.5% ethanol / 2.5% Tween 80 / 95% saline) (Ethanol, Suzhou TruwayBio) (Tween 80, WXBC8472V, Suzhou TruwayBio) (saline, 10B19091603, Suzhou TruwayBio). For H358, H441 and A549 xenograft models, tunlametinib single drug treatment groups were treated with tunlametinib (0.5 mg/kg PO, BID or 3.5 mg/kg BIW). The combination drug groups in H358 xenograft were treated with docetaxel (10 mg/kg, QW × 2 weeks, 7.5 mg/kg, QW × 1 week and 5 mg/kg, QW × 1 week). The doses of docetaxel group in H441 xenograft model were set at 10 mg/kg (QW × 2 weeks) followed by 7.5 mg/kg (QW × 2 weeks) while in A549 xenograft model, the dose of docetaxel was set at 10 mg/kg (QW × 1 week) and 7.5 mg/kg (QW × 4 weeks), the combination drug treatment groups received with their respective dose administration.

**Reference**

1. Mosmann, T., Rapid Colorimetric Assay for Cellular Growth and Survival: Application to Proliferation and Cytotoxicity Aasays. *Journal of Immunological Methods* **1983,** 65, 55-63.

**Supplementary Table S1. *In vivo* efficacy study information**

| **Figure ID.** | **Tunlametinib therapy** | **CDX and PDX model** | **Derivation** | **Cell numbers** | **Mice species** | **Animal vendors** |
| --- | --- | --- | --- | --- | --- | --- |
| Figure 3-A | Tunlametinib monotherapy | 1^st^A375 xenograft | Tumor fragment | -- | BALB/c nude | Beijing HFK Bioscience Co., Ltd |
| Figure 3-B | Tunlametinib monotherapy | 1^st^Colo205 xenograft | Tumor fragment | -- | BALB/c nude | Beijing HFK Bioscience Co., Ltd |
| Figure 3-C | Tunlametinib monotherapy | HT29 xenograft | Tumor fragment | -- | BALB/c nude | Beijing HFK Bioscience Co., Ltd |
| Figure 3-D | Tunlametinib monotherapy | 1^st^ Calu 6 xenograft | Tumor fragment | -- | BALB/c nude | Beijing HFK Bioscience Co., Ltd |
| Figure 3-E | Tunlametinib monotherapy | NCI-H1975 xenograft | Cell line | 2.5×10^6^ | BALB/c nude | Beijing HFK Bioscience Co., Ltd |
| Figure 3-F | Tunlametinib monotherapy | 2^nd^A375 xenograft | Cell line | 5×10^6^ | BALB/c nude | SPF(Beijing)Biotechnology Co., Ltd |
| Figure 3-G | Tunlametinib monotherapy | 2^nd^ Colo205 xenograft | Cell line | 5×10^6^ | BALB/c nude | SPF(Beijing)Biotechnology Co., Ltd |
| Figure 3-H | Tunlametinib monotherapy | CR0004 | Tumor fragment | -- | BALB/c nude | Beijing Anikeeper Biotech Co., Ltd |
| Figure 3-I | Tunlametinib monotherapy | CR0029 | Tumor fragment | -- | BALB/c nude | Beijing Anikeeper Biotech Co., Ltd |
| Figure 3-J | Tunlametinib monotherapy | CR2179 | Tumor fragment | -- | BALB/c nude | Beijing Anikeeper Biotech Co., Ltd |
| Figure 3-K | Tunlametinib monotherapy | CR6289 | Tumor fragment | -- | BALB/c nude | Beijing Anikeeper Biotech Co., Ltd |
| Figure 5-A | Tunlametinib+SHP2 inhibitor | H358 xenograft | Cell line | 5×10^6^ | NOD-Scid | Shanghai Lingchang Bio Tech Co., Ltd |
| Figure 5-B | Tunlametinib+KRAS^G12C^ inhibitor | H358 xenograft | Cell line | 5×10^6^ | NOD -Scid | Shanghai Lingchang Bio Tech Co., Ltd |
| Figure 5-C | Tunlametinib +BRAF inhibitor | 3^rd^ A375 xenograft | Tumor fragment | -- | BALB/c nude | Shanghai SLAC Laboratory Animal Co., Ltd |
| Figure 5-D | Tunlametinib + docetaxel | 2^nd^ Calu 6 xenograft | Cell line | 5×10^6^ | BALB/c nude | Vital River Laboratory Animal Technology Co., Ltd. |
| Figure 5-E | Tunlametinib + docetaxel | H358 xenograft | Cell line | 5×10^6^ | NOD -Scid | Shanghai Lingchang Bio Tech Co., Ltd |
| Figure 5-F | Tunlametinib +docetaxel | H441 xenograft | Cell line | 5×10^6^ | NU/NU mice | Shanghai Lingchang Bio Tech Co., Ltd |
| Figure 5-G | Tunlametinib + docetaxel | A549 xenograft | Cell line | 5×10^6^ | BALB/c nude | Zhejiang Vital River Laboratory Animal Technology Co., Ltd |

**Supplementary Table S2. Selectivity of tunlametinib at 10 μM on the inhibitory activity against 77 kinases *in vitro***

| **Kinase** | **Testing method** | **Inhibition rate（%）** | **Kinase** | **Testing method** | **Inhibition rate（%）** |
| --- | --- | --- | --- | --- | --- |
| Abl kinase *(h)* | LANCE | 6.9 | JAK2 *(h)* | LANCE | 12.8 |
| ALK *(h)* | LANCE | -0.3 | JAK3 *(h)* | LANCE | 3.1 |
| Akt1/PKBα *(h)* | LANCE | 2.2 | JNK1 *(h)* | LANCE | 1.9 |
| Akt2/PKB β *(h)* | LANCE | -3.7 | JNK2 *(h)* | LANCE | 9.9 |
| Akt3/PKB γ *(h)* | LANCE | -1.3 | JNK3 *(h)* | LANCE | 3.4 |
| ALK4 *(h)* (ACVR1B) | LANCE | -3.8 | KDR kinase *(h)* (VEGFR2) | LANCE | 7.4 |
| AurA/Aur2 kinase *(h)* | LANCE | 5.7 | Lck kinase *(h)* | LANCE | -5.3 |
| AurC/Aur3 kinase *(h)* | HTRF | 2.7 | LTK *(h)* | LANCE | 4 |
| Axl kinase *(h)* | LANCE | -0.9 | Lyn A kinase *(h)* | LANCE | 6.1 |
| CHK1 *(h)* | LANCE | 5.2 | Lyn B kinase *(h)* | HTRF | 18.2 |
| CHK2 *(h)* | LANCE | 6.6 | MEK5 *(h)* (MAP2K5) | LANCE | 3.8 |
| c-kit kinase *(h)* | LANCE | 1.5 | MEK1/MAP2K1 *(h)* | LANCE | 100.5 |
| c-Met kinase *(h)* | LANCE | 22.9 | Mer kinase *(h)* | LANCE | 4.7 |
| DDR2 kinase *(h)* | LANCE | 0.3 | MKK6 /p38α (*h*) | HTRF | 5.1 |
| EGFR kinase *(h)* | LANCE | 10.4 | MusK *(h)* | LANCE | 5.1 |
| EphA1 kinase *(h)* | HTRF | -13.1 | p38α kinase *(h)* | LANCE | -1.1 |
| EphA2 kinase *(h)* | LANCE | -2.4 | p38β2 kinase *(h)* (SAPK2b2) | HTRF | -9.2 |
| EphB1 kinase *(h)* | LANCE | -4.1 | p70S6K *(h)* | LANCE | -10.9 |
| EphB2 kinase *(h)* | LANCE | 7.1 | p70S6Kβ *(h)* | LANCE | 4.4 |
| ERK_1_ *(h)* | LANCE | -4.9 | PDGFRα kinase *(h)* | LANCE | 0.9 |
| ERK_2_ *(h)* (P42^mapk^) | LANCE | -4.5 | PDGFRβ kinase *(h)* | LANCE | -3.9 |
| ERK5 *(h)* (MAPK7) | LANCE | -2.9 | Pim1 kinase *(h)* | LANCE | 47.5 |
| FAK *(h)* | LANCE | 7.7 | Pim2 kinase *(h)* | LANCE | 4.5 |
| FGFR1 kinase *(h)* | LANCE | 6.3 | PKA *(h)* | LANCE | 5.3 |
| FGFR2 kinase *(h)* | LANCE | 2.3 | PKCα *(h)* | HTRF | 1.8 |
| FGFR3 kinase *(h)* | LANCE | -3.6 | Raf-1 kinase *(h)* | LANCE | 13.9 |
| FGFR4 kinase *(h)* | LANCE | -6.2 | Ret kinase *(h)* | HTRF | 8.9 |
| FLT-1 kinase *(h)* (VEGFR1) | LANCE | 2 | ROCK1 *(h)* | LANCE | -1.3 |
| FLT-3 kinase *(h)* | LANCE | -1 | ROCK2 *(h)* | LANCE | -1.8 |
| FLT-4 kinase *(h)* (VEGFR3) | LANCE | 13.2 | Ron kinase *(h)* | LANCE | 0.3 |
| Fms/CSFR kinase *(h)* | LANCE | 7.6 | Src kinase *(h)* | LANCE | 18.6 |
| GSK3α *(h)* | LANCE | 6.8 | TIE2 kinase *(h)* | LANCE | -14.2 |
| GSK3β *(h)* | LANCE | 3.3 | mTOR kinase *(h)* (FRAP1) | LANCE | -4.1 |
| HER2/ErbB2 kinase *(h)* | HTRF | -16.1 | TRKA *(h)* | LANCE | 2.4 |
| HER4/ErbB4 kinase *(h)* | HTRF | 7.4 | TRKB *(h)* | LANCE | 0.3 |
| IGF1R kinase *(h)* | LANCE | -15.8 | TRKC *(h)* | LANCE | -3.5 |
| IRK *(h)* (InsR) | LANCE | -4.4 | Tyk2 *(h)* (JTK1) | LANCE | 8 |
| IRR kinase *(h)* | LANCE | -4.8 | Tyro3 /Sky kinase *(h)* | LANCE | 22.8 |
| JAK1 *(h)* | LANCE | 4.1 |  |  |  |

**Supplementary Figure S1. Experimental design of in vivo studies.**

**
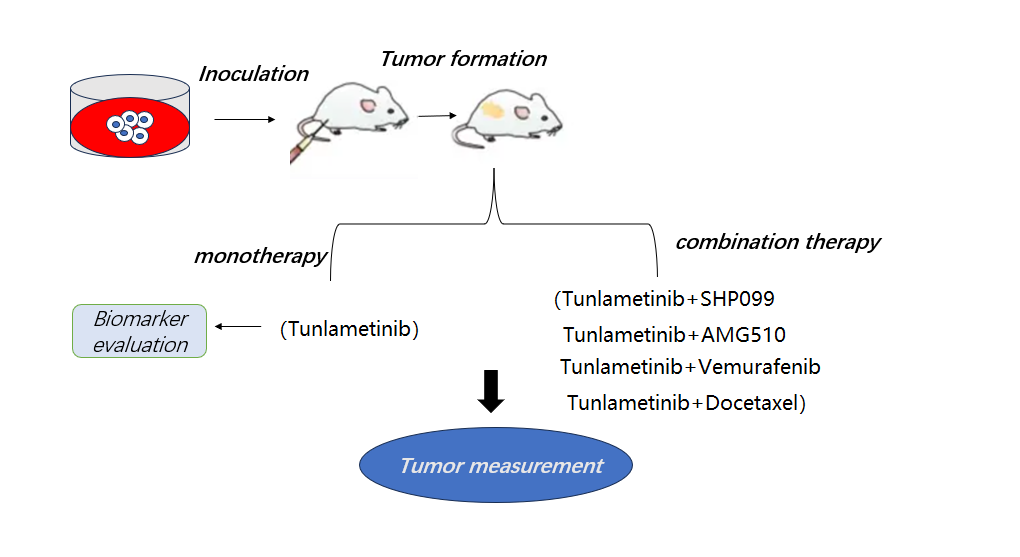
**

**Supplementary Figure S2.** **Anti-proliferative dose-response curves of tunlametinib, AZD6244 and GSK212 on 11 tumor cell lines and 1 human embryonic lung cells (MRC-5) *in vitro.***


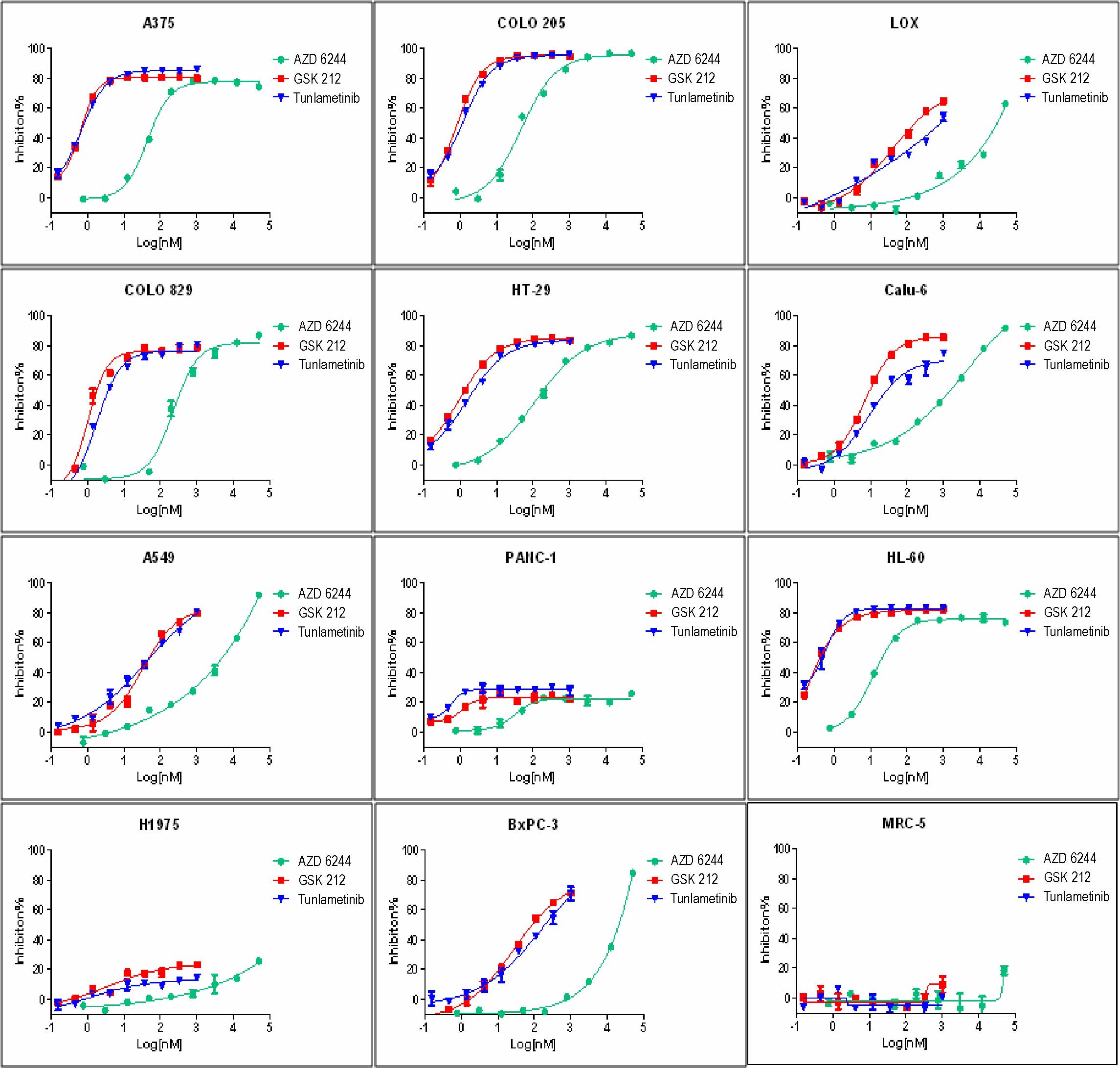
**Supplementary Figure S3. Mice body weights of tunlametinib as monotherapy in CDX or PDX models. A**, **C, D,** A375, HT-29, Calu-6 CDX_S_ were treated with vehicle, tunlametinib (1, 3, 6 mg/kg, QD) or AZD6244 (25mg/kg, BID). **B,** COLO 205 CDX was treated with vehicle, tunlametinib (1, 3, 9 mg/kg, QD) or AZD6244 (50 mg/kg, BID). **E,** NCI-H1975 CDX was treated with vehicle, tunlametinib (3, 9 mg/kg, QD) or docetaxel (10 mg/kg, QW). **F, G** A375, COLO 205 CDXs were treated with vehicle, tunlametinib (1, 3 mg/kg) or MEK162 (10 mg/kg, QD). **H-K,** CR0004, CR0029, CR2179, CR6289 PDXs were treated with vehicle and tunlametinib (1 mg/kg). For A375 CDX model, N=14 mice in vehicle group, and N=7 in tunlametinib and AZD6244 treatment groups. For other CDX models, N=8 mice in each group. For PDX model, N=2 mice in vehicle group, and N=3 mice in tunlametinib treatment groups. Data was shown as mean ± SEM.


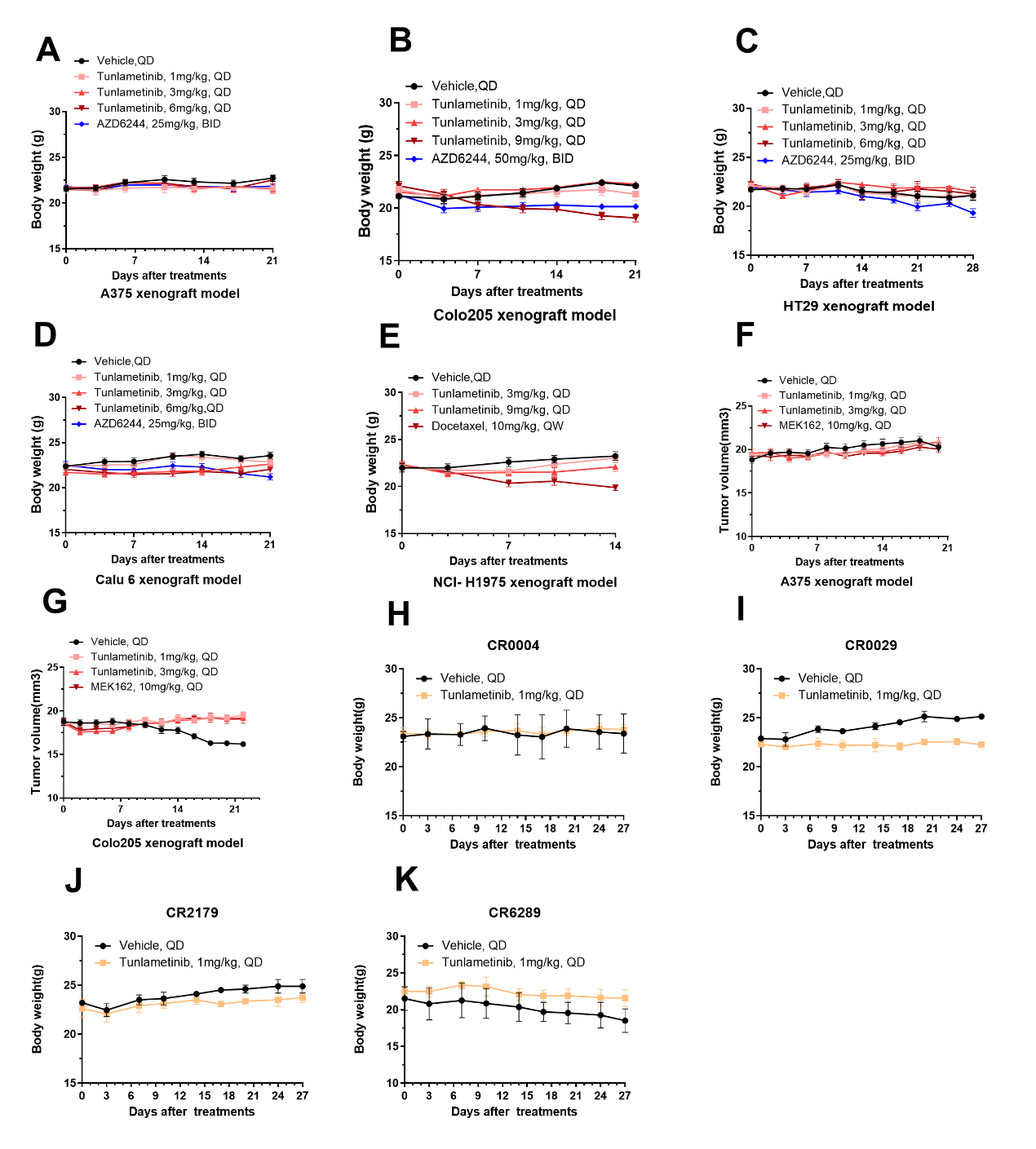


**Supplementary Figure S4.** **Mice body weights of tunlametinib as combination treatment in CDX model.** **A**, Tunlametinib combined with SHP099 in H358 xenograft model (N=8 per group). **B**, Combination of tunlametinib and AMG510 (N=8 per group). **C**, Tunlametinib in combination with vemurafenib (N=6 per group). **D-G,** Tunlametinib combined with docetaxel in KRAS mutant xenograft models (Calu-6, H358, H441 and A549 xenograft models) (N=8 per group). Data was shown as mean ± SEM.


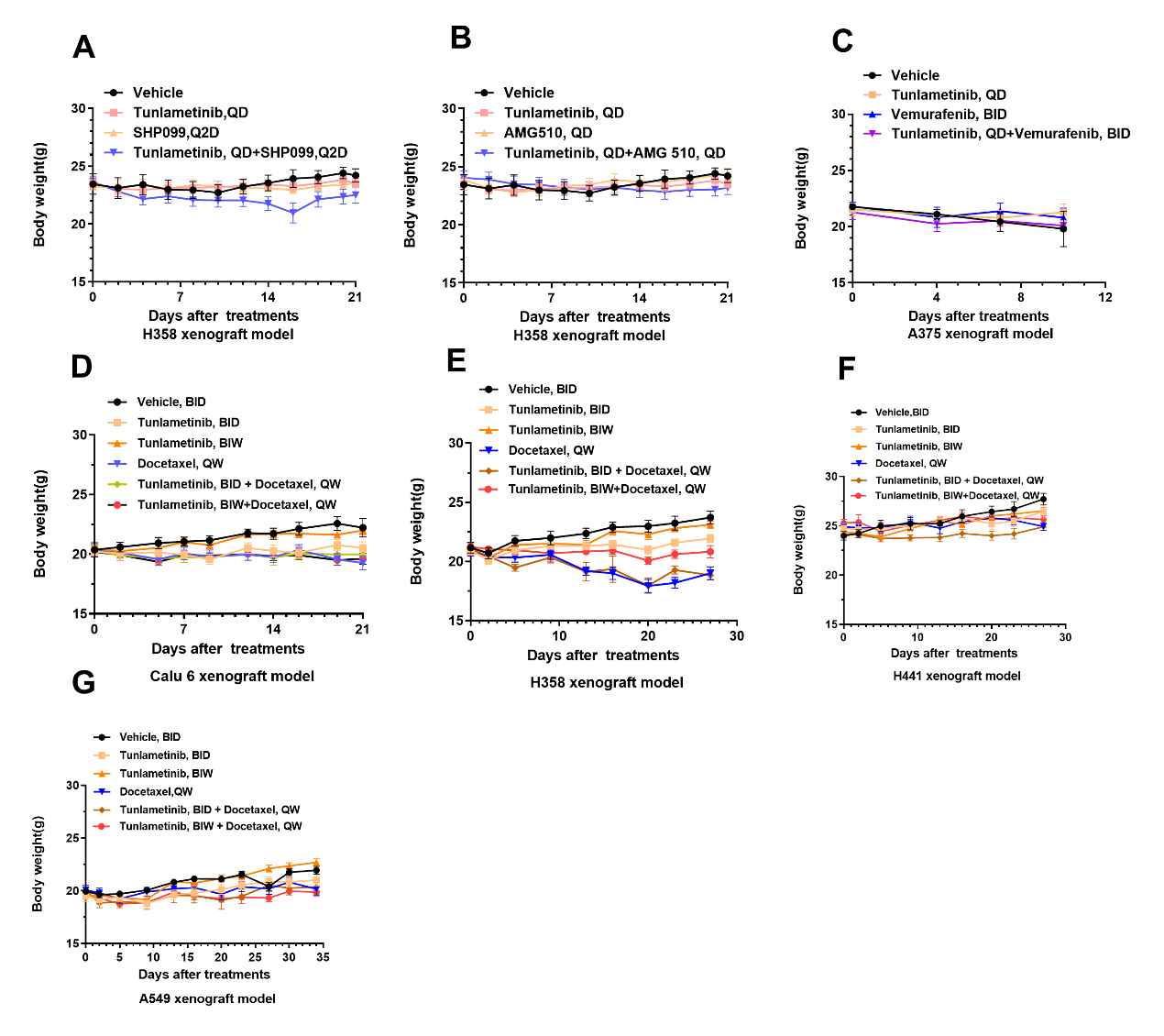

Supplement: Supplementary file 1 [file DataSheet1.docx]
